# A Survey on Data Quality for Dependable Monitoring in Wireless Sensor Networks

**DOI:** 10.3390/s17092010

**Published:** 2017-09-02

**Authors:** Gonçalo Jesus, António Casimiro, Anabela Oliveira

**Affiliations:** 1 Hydraulics and Environment Department, LNEC, Lisbon 1700-066, Portugal; 2 LaSIGE, Faculdade de Ciências, Universidade de Lisboa, Lisbon 1749-016, Portugal

**Keywords:** wireless sensor networks, dependability, machine learning, monitoring, data quality, sensor fusion

## Abstract

Wireless sensor networks are being increasingly used in several application areas, particularly to collect data and monitor physical processes. Non-functional requirements, like reliability, security or availability, are often important and must be accounted for in the application development. For that purpose, there is a large body of knowledge on dependability techniques for distributed systems, which provide a good basis to understand how to satisfy these non-functional requirements of WSN-based monitoring applications. Given the data-centric nature of monitoring applications, it is of particular importance to ensure that data are reliable or, more generically, that they have the necessary quality. In this survey, we look into the problem of ensuring the desired quality of data for dependable monitoring using WSNs. We take a dependability-oriented perspective, reviewing the possible impairments to dependability and the prominent existing solutions to solve or mitigate these impairments. Despite the variety of components that may form a WSN-based monitoring system, we give particular attention to understanding which faults can affect sensors, how they can affect the quality of the information and how this quality can be improved and quantified.

## 1. Introduction

In order to increase the dependability of monitoring applications in wireless sensor network (WSN) settings, one must be aware that the quality of monitoring data can be affected by faults. In essence, there is a problem of data quality assurance, which can be faced taking two main perspectives: either by deploying dependability techniques to mask faults and enforce the reliability of the system or by enhancing the system with means to continuously assess and characterize the quality of data [1]. In the former case, the system will not be aware of the quality of data, and hence, if a certain quality is needed, it must be enforced by design, confining the effects of faults a priori. Considering sensors to be the main source of data, errors in sensing measurements are handled by procedures that are established based on a deep understanding of the characteristics of the sensors [2]. Missing readings may be handled by oversampling, and glitches, like outliers and noise, can be masked by averaging. In the latter case, given that the system can be aware of the quality of data at run-time, it is better suited to be used in environments where full knowledge of the operational conditions is not known in advance. In this case, mitigation techniques must be deployed to handle faults and data quality problems at run-time, for instance exploiting application semantics to determine appropriate data corrections and to regain the needed data quality. Given that no system can be built to exhibit 100% reliability, the two perspectives can be combined. In this paper, we take the latter perspective and consider that the quality of sensor data can be assessed, providing an indication of the overall system health, encompassing sensors, the wireless network and the processing tasks.

Assuring the quality of sensor data for a dependable operation is particularly challenging in some WSN-based monitoring applications. In fact, it is often the case that the sensors and the WSN are deployed in harsh environments and exposed to extreme physical conditions, thus being more likely affected by faults. The problem becomes critical when dependability is an important application requirement. For instance, in water-related information systems, inaccurate information in aquatic monitoring may lead to false warnings being issued or harmful situations not being detected early enough (e.g., floods or pollution events). As another example, WSNs are deployed in data centers for flexible temperature monitoring and energy-efficient control of air-cooling equipment [3,4]. Therefore, ensuring the accuracy of collected data is also necessary for effectiveness reasons. In these examples, the operational conditions are typically hard to accurately predict, ensuring that the reliability of operations is often hard or costly, and the consequences of inaccurate sensor data collection can be severe.

In this survey, we characterize and systematize existing solutions to dependable monitoring in WSNs by approaching them in two steps. In the first step, we look at the root cause of dependability problems concerning the quality of sensor data, that is we identify and analyze several kinds of faults that may affect the system operation, in particular at the sensor and network levels, describing the specific effect on the sensor data and the relevant failure modes [5] that allow abstracting particular kinds of faults. When appropriate, we also refer to particular mitigation solutions to automatically adjust the sensors measurements according to each disturbance. Then, we provide a comprehensive overview of the solutions to achieve improved sensor data quality and dependable operation of WSN-based monitoring applications. In addition to detection and correction strategies, fault-tolerance strategies based on sensor data fusion procedures, exploiting the availability of redundant measurements or available modeling surrogates, are surveyed. However, there is a focus on works and solutions related to monitoring in aquatic environments, noting that these solutions are also applicable in many other contexts, but that the opposite might not be true, in particular concerning solutions that are not agnostic to the semantics of the monitored data.

The remainder of the paper is organized as follows. Section 2 exposes a set of fundamental ideas related to the issue of data quality in WSNs, motivating the need for dependability, introducing the baseline architecture for a WSN-based monitoring system and referring to the main dependability strategies that may be employed. Section 3 describes the notion of data quality and the main aspects that may affect this quality during monitoring. Section 4 presents an overview of solutions for dependable sensor networks. We will conclude in Section 5, with a discussion of the possible results of the solutions mentioned herein.

## 2. Overview of Key Issues

### 2.1. Motivation

Complex and powerful forecast systems are now able to predict environmental variables such as storm events with small errors, but they depend on a continuous flow of confirmation with real-time data for robustness. Real-time monitoring data, such as surface water elevation, flow or water quality variables depend solely on the sensor hardware deployed in the physical environment (oceans, river, lakes, etc.) and its proper maintenance.

The effectiveness of existing emergency warning and forecast procedures for natural and man-made hazardous events may be limited by several factors, including an often sparse and unreliable real-time observational network, the use of coarse-resolution prediction models and the reliance on traditional approaches to convey warning and forecast information.

In spite of the vast research on the dependability of distributed systems, in particular on computational architectures/frameworks for reliable and timely operations, monitoring systems pose new challenges to dependability. The sensory and sensor network technologies, which are now becoming widely available, are subject to diverse hazards and are not sufficiently reliable and robust against harsh exogenous and/or environmental factors. In this field, there is still a lack of architectural, fault-tolerant and system management solutions, which are essential for dependable, robust remote monitoring, necessary for adequate water management.

Ensuring the quality of monitoring data is fundamental to avoid false alarms or ignoring relevant data. However, because these sensors are located in the physical environment, they are constantly being subjected to factors that directly interfere with the data quality, such as potentially strong currents, debris accumulation and tough weather conditions. Consequently, there is a trust issue related to the collected data, which demands an extensive human intervention in terms of time and knowledge specialization, data validation tasks and periodic maintenance of sensors. To deal with this problem, it is necessary to continuously and automatically characterize the quality of collected data. Hence, the application of techniques based in the existence of redundancy at the data collection and data processing levels is a promising approach.

### 2.2. Monitoring and Data Processing

The process of environment monitoring requires sensor devices to be deployed within the system. These sensors will be the entities responsible for measuring the parameters of interest, like temperature, water level or salinity. A sensor essentially converts a physical quantity in its input to an electrical signal, produced as the output, which is usually proportional to the input. Further to the sensor itself, additional components are needed to perform signal processing functions, store measured values and communicate these values to other systems. It is hence usual to refer to these more complex components as smart sensors or intelligent sensors [6], typically interconnected to other smart sensors to form wireless sensor networks.

For monitoring and control purposes, wireless sensor networks have become a subject of interest in recent years, mostly due to enormous advances in sensing and communication technology, which has fostered the use of smart sensors, with applications in many fields: (a) military applications; (b) environmental monitoring; (c) commerce; (d) human-centric applications; and (e) applications to robotics (Arampatzis et al. [7]). WSNs are formed by smart sensor nodes, and each sensor node may have several individual sensors, in which case they are said to be clustered. Information collected by sensor nodes is typically transmitted to a sink or controller node.

**Figure 1:**
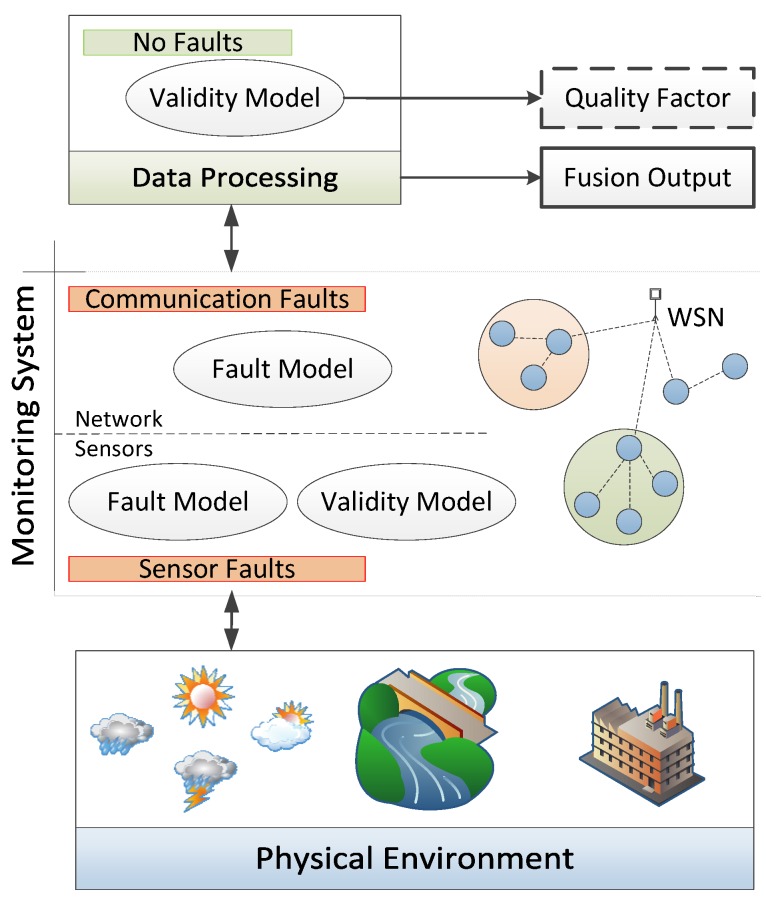
Generic view of the WSN-based monitoring system.

After the sensors measurement step, performed usually through a WSN, there is another layer in the overall system that comprises the gathering and analysis of the measurements, including information fusion (Section 4). This phase is commonly referred to as data processing (see Figure 1).

Our goal is to describe and enumerate the processes involved in each layer of the scheme in Figure 1. In a bottom-up perspective, the first layer is the physical environment (it can also be defined as the object or the event to be monitored), which can have a great influence on the measurements. Although there are many specific external factors related to perturbation events or objects in all of the different applications of WSNs, we decided to tackle the problems of the involved water body by first enumerating well-known limitations of sensor devices in Section 3.2.

**Figure 2:**
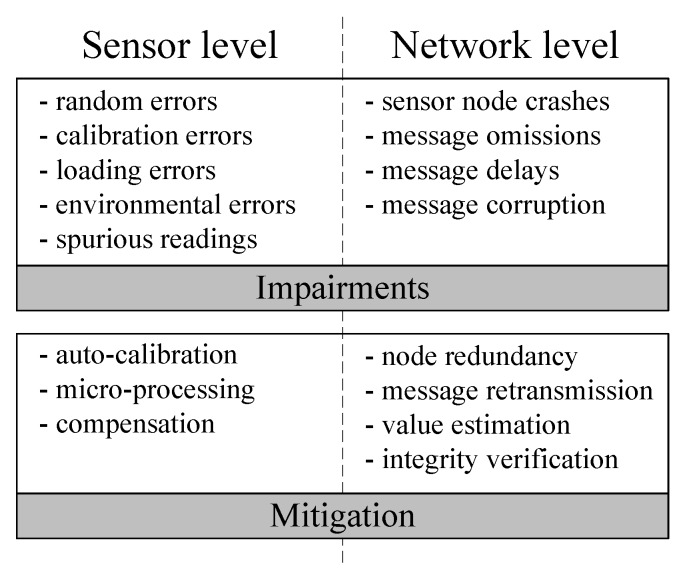
Sensor and WSN faults and mitigation solutions.

On the second layer, we are considering that in terms of faults and validity issues, monitoring has two abstraction levels: the sensors and communication between them. Each level has a particular fault model, with faults arising from different sources (see Figure 2). We will explore these subjects in Section 3.2 and Section 3.3 with the respective mitigation solutions.

Finally, in order to centrally analyze all of the sensing information, a third layer appears in the system. In the data processing layer, it is possible to infer the quality of the gathered information, through fusion processes of redundant and related measurements, by multi-sensor fusion methods, or by expert-knowledge of the system model. In fact, this is where we can ultimately handle both sensor and network-level problems and apply the mitigation techniques identified already in Figure 2. These techniques will be addressed in Section 4.

### 2.3. Dependability Strategies

When designing a fault-tolerant and dependable system, the typical means to deal with system errors and faults include error detection and error or fault recovery. In this context, endowing the system with redundant components can be instrumental to compensate existing errors or faults affecting some component. The affected component can be replaced in its tasks by the spare, redundancy component, which will ensure that the system function will continue to be provided. However, redundancy does not refer solely to having multiple similar components, which is a form of space redundancy. It is also possible to implement forms of redundancy in the time (e.g., repeating some action multiple times) and in the value domains (e.g., adding extra information) [8].

Some examples of space redundancy include storing information in several disks, machines or data centers, having multiple nodes performing the same computation (either in parallel, called active replication, or with some nodes in stand-by mode, called passive replication), or sending a network message through multiple network paths. Time redundancy is typically explored in reliable communication systems that retransmit messages when they suspect that these messages might have been lost in previous transmissions. Restarting an aborted transaction or a deadlocked computation are also examples of time redundancy. Finally, value redundancy is observed in data storage and communication systems that use error correcting codes associated with the stored or transmitted data, allowing the original information to be reconstructed when some bits or parts of the information become corrupted. To deal with malicious forms of information corruption, cryptographic signatures may be used.

In the application of these concepts to sensor validation, [9] stated that there are two classical approaches that are widely used: (a) analytical redundancy and (b) hardware redundancy. Analytical redundancy uses mathematical relationships between measurements to predict or infer a sensor’s value. Two disadvantages of this approach are the possible inefficiency of the mathematical processes when we have a large number of sensors and the model complexity increases and the fact that the mathematical relationships can be very data specific, and a slight modification may require significant efforts to stabilize. Hardware redundancy is not always possible because of the costs implied by additional sensors and their installation and maintenance operations.

The right approach to be used depends on several issues, like the assumed fault model, the criticality of the application, the cost or timeliness requirements. In some cases, several dependability techniques can be used in a single system to deal with different problems or to achieve the needed levels of assurance. This is particularly true in complex systems, like aquatic systems, in which different techniques can be applicable to mitigate faults in the sensing process and to handle WSN faults. Combinations of the solutions mentioned later in this survey (Section 4.1) may thus be used in the design of a single system.

## 3. Sensor Data Quality

When the quality of sensor data is an important attribute for the dependability of the application, it becomes necessary to somehow express this quality, which can be done in various ways. Additionally, a priori knowledge about the possible causes of quality degradation, translated into faults and a corresponding fault model, is also relevant. It will enable a more accurate characterization of the quality of sensor data and the possibly of incorporating in the system some techniques to mitigate the effects of specific faults assumed in the fault model. These aspects are addressed in the following sections.

### 3.1. Expressing Data Quality

The interpretation and modeling of the available information into adequate theoretical frameworks is the main means to characterize the quality of the obtained sensor data. These qualitative interpretations of sensor data can become confusing when different authors introduce an array of terms for quality (the most generic), including:

- Validity is typically employed when a determined requirement about the quality of data is available, against which it is possible to compare some quality measure and declare if the data are valid [1,10].
- Confidence is an attribute that may be elaborated from the continuous observation of sensor data, without the need for a quality requirement to be available. It is generally used when datasets are available and can be characterized in a probabilistic way, along with model fitting or threshold definition techniques, to yield continuous or multi-level confidence measures [11].
- Reliability is a typical dependability attribute [12], expressing the ability of a system to provide the correct service (or the correct data, for that matter) over a period of time. The term data reliability in sensor networks is often considered when transmissions and/or communications may be subject to faults like omissions or a total crash [13,14].
- Trustworthiness is mostly employed in connection with security concerns, namely when it is assumed that data can be altered in a malicious way. In the context of sensor networks, it characterizes the degree to which it is possible to trust that sensor data have not been tampered with and have thus the needed quality [15].
- Authenticity is also used, in particular in a security context, but to express the degree to which it is possible to trust the claimed data origin [16]. This is particularly important when the overall quality of the system or application depends on the correct association of some data to their producer.

This terminology does contain other terms, including other aspects of data quality that are implicit and briefly approached herein, such as timeliness, precision, tunability, completeness, usability, accuracy, throughput, affordability and reusability [17]. We will also describe herein the diverse typologies of data quality and how to obtain a quality parameter, either for each individual sensor or for the global system, according to several studies. Therefore, in terms of applicability, we must differentiate single-sensor validity from multi-sensor fusion validity, when several sensors exist and sensor fusion can be applied.

In single-sensor situations, there are models or related information that allow reasoning about an individual sensor’s data quality without requiring other sensors’ data. The work in [18] tried to identify faulty situations (see Section 3.2) such as noise and outliers in chlorophyll concentration sensors deployed in lake water, by implementing different fault detection methods:

- Rule-based methods that use expert knowledge about the variables that sensors are measuring to determine thresholds or heuristics with which the sensors must comply.
- Estimation methods that define a “normal” behavior by considering spatial and temporal correlations from sensor data. A sensor reading is matched alongside its forecasted value to assess its validity.
- Learning-based methods that define models for correct and faulty sensor measurements, using collected data for building the models.

In the example from [18], all three methods were used to assert the correctness (or incorrectness) of the collected data, thus adopting a Boolean approach to quality characterization. However, the same methods may be employed in other ways, as a means to characterize quality in a step-wise or even continuous way. For instance, and still considering a single-sensor situation, [11] employed fuzzy logic rules to obtain a qualitative sense of a sensor’s validity based on its own historical behavior represented by a confidence measure.

In a multi-sensor situation, the quality of sensor measurements is characterized by using redundant or correlated data obtained from the different sensors. This redundancy allows for data fusion methods to be deployed at the network level, resulting in improved (fused) sensor data, as well as improved data quality characterization. Various quality-oriented network meta-models can be explored according to the application requirements. For instance, in [17], the data quality is calculated through the several nodes (and the sink) on the entire WSN structure.

Sensor data fusion methods will be detailed in Section 4. As for the quality characterization process when data fusion is performed, the applicable methodology depends on the available information concerning the quality of individual sensor measurements. In fact, this information can also be used in the data fusion process itself.

For instance, in [11], the approach relied on a statistical method (Parzen estimation of the probability density function) to determine the variance of sensors’ data and to calculate the average of the sensors, considering just the sensors with a high-quality standard in the data fusion process. If all sensors are producing high-quality data, then the fusion will also reach the highest possible quality. Otherwise, better results will be achieved when discarding sensor data with lower quality, rather than using these data in the fusion process.

Another example can be found in [19], where reliability estimates are calculated for sensor data, using Bayesian networks or random forests to obtain reliability coefficients, and then, these reliability estimates are used in a sensor fusion process to discard sources that are considered unreliable if the reliability estimate is below a defined threshold.

Regarding the quantification of data quality, the two main approaches consist of considering discrete quality classes or continuous quality values.

In the discrete approach, it is possible to use binary classes, such as {valid,invalid} [20], or to use a multi-level class, like {verylow,low,high,veryhigh} [11]. These discrete classifications can be applied to each sensor (individual sensor data) or to the whole network of sensors (fused data).

In the continuous approach, a confidence level is usually derived, ranging in a well-defined continuous interval (often [0,1] or equivalently [0%,100%]). Therefore, the validity of sensor information may not only have the values “true and” “false”, especially if one must process continuously-valued data [1,9]. For instance, a noisy sensor (internal or external noise) may deliver useful data within some error margin, but the quality of that data is lower than that from a non-noisy sensor. In a multi-sensor fusion application, the quality quantification can be calculated using a cumulative association of each sensor quality coefficient [21] or calculating the percentage of sensors used in the fusion against the sensors in the network.

### 3.2. Sensor Level Faults

In this subsection, we present a systematization of the main types of sensors and their characteristics, classifying the various data errors that may be produced by sensors. From the perspective of building modular dependable systems, what is interesting is to group the several possible faults and the consequent data errors into well-defined sensor failure modes. We thus identify the relevant failure modes under which a sensor can fail and produce data with degraded quality. The focus herein is on the sensor level, whereas the next subsection addresses network level faults. Finally, we also focus on possible mitigation techniques to handle sensor faults.

#### 3.2.1. Sensor Characteristics

We begin to dissect sensor faults by exploring the transducing processes, enumerating the different methods to convert the various physical effects into electric signals, as well as each one’s advantages and limitations. This enumeration is important to the survey, to understand the most basic origins of faults in sensors. The sensor material characteristics or the harshness of the environmental conditions lead to the production of a specific kind of fault. Some sensors strive to perceive an object that is moving in dusty environments, while others experience issues reading a correct level observation in fluids. For instance, capacitive sensors present a considerable sensitivity and require low energy usage, making them an attractive choice for many areas, but as pointed out by [22], the response characteristics of these sensors are very nonlinear, and the offset capacitance is non-negligible and must be handled to correctly detect capacitance variations due to the applied pressure and to avoid errors. In summary, from a dependability perspective, it is important to distinguish sensors in terms of their operation and robustness to distinct environment conditions. When a sensor is highly sensitive, but frequently faulty, a redundancy solution must be considered, possibly using a sensor that offers the same sensitivity, but is more reliable.

The main types of sensors according to the exploitation of displacement effects are the following [23]:

- Resistance: Resistive sensors, also termed potentiometers, are based on an electromechanical instrument that transforms a mechanical variation, like a displacement, into an electrical signal capable of being monitored following conditioning;
- Induction: Inductive sensors are primarily based on the principles of magnetic circuits and may be categorized as self-generating or passive;
- Capacitance: Capacitive sensors depend on variations in capacitance in reply to physical changes. A capacitive level pointer uses the changes in the comparative permittivity among the plates;
- Piezoelectricity: Piezoelectricity is the term used to determine the capacity of specific materials to create an electric charge that is relative to a directly applied mechanical pressure;
- Laser: Laser sensors compare changes in optical path length and in the wavelength of light, which can be determined with very little uncertainty. Laser sensors achieve a high precision in the length and displacement measurements, where the precision achieved by mechanical means is not enough;
- Ultrasonic: Uses the time-of-flight method as the standard for the use of ultrasound for monitoring purposes. A pulse of ultrasound is transmitted in a medium, reflecting when it reaches another medium, and the time from emission to recognition of the reflected pulsation is read;
- Optical: Optical sensors encompass a variety of parts that use light as the means to convert kinetics into electrical signals, comprised mostly of two components: a main diffraction grating, representing the measurement standard (scale); and a detection system. What is detected is the position of one regarding the other;
- Magnetic: A magnetic sensor is either triggered to function by a magnetic field or the use of the field that defines the properties of the sensor;

In Table 1, a summary of the relative advantages and disadvantages of each of the described displacement effects is presented. The goal here is not to choose the best type of sensor, but to discriminate the strong and weak points of all of the types.

Beyond the limitations of the transducers, [26] explained other causes of measurement uncertainty and how only an estimation of the observed physical property can be given. When considering individual sensor measurements, the possible types of errors observed in measurement values can be classified as follows:

- Random errors are described by an absence of repeatability in the readings of the sensor, for instance due to measurement noise. These errors tend to happen on a permanent basis, but have a stochastic nature;
- Systematic errors are described through consistency and repeatability in the temporal domain. There are three types of systematic errors at the sensor level:
  - Calibration errors result from errors in the calibration procedure, often in relation to linearization procedures;
  - Loading errors emerge when the intrusive nature of the sensor modifies the measurand. Along with calibration errors, loading errors are caused by internal processes;
  - Environmental errors emerge when the sensor experiences the surrounding environment and these influences are not considered. In contrast with the previous two types of errors, environmental errors are due to external factors;
- Spurious readings are non-systematic reading errors. They occur when some spurious physical occurrence leads to a measurement value that does not reflect the intended reality. For instance, a light intensity measurement in a room can provide the wrong value if obtained precisely when a picture of the room is taken and the camera flash is triggered.

#### 3.2.2. Sensor Failure Modes

The classification presented above builds essentially on the persistence and nature of the observable value errors. An alternative way to acknowledge and to deal with the fact that sensor measurements are affected by uncertainties, which is commonly used when building modular distributed systems, is to identify relevant sensor failure modes. Independently of the several factors leading to a sensor fault and the consequent measurement error(s), the faulty behavior of the sensor component is observed through its interface, that is, through the values it produces. Therefore, a failure mode characterizes a certain deviating behavior, abstracting its causes and considering only the measurement values produced at the sensor interface.

**Figure 3:**
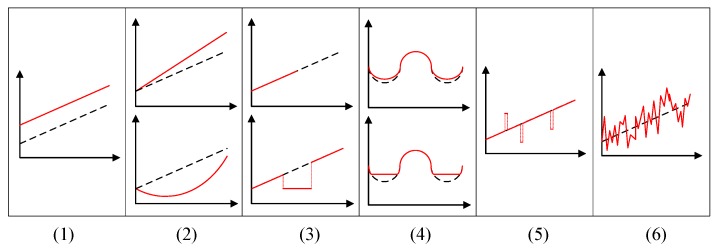
Sensors’ failure modes. The faulty sensor output is represented with a filled line, whereas the real values are depicted with a dashed line.

The main sensor failures modes, depicted in Figure 3, are the following [1]:

1. Constant or offset failure mode: The observations continuously deviate from the expected value by a constant offset.
2. Continuous varying or drifting failure mode: The deviation between the observations and the expected value is continuously changing according to some continuous time-dependent function (linear or non-linear).
3. Crash or jammed failure mode: The sensor stops providing any readings on its interface or gets jammed and stuck in some incorrect value.
4. Trimming failure mode: The observations are correct for values within some interval, but are modified for values outside that interval. Beyond the interval, the observation can be trimmed at the interval boundary or may vary proportionally with the expected value.
5. Outliers failure mode: The observations occasionally deviate from the expected value, at random points in the time domain;
6. Noise failure mode: The observations deviate from the expected value stochastically in the value domain and permanently in the temporal domain.

Comparing this classification of sensor failure modes with the classification of sensor errors previously introduced, it is interesting to note the direct correspondence between the class of random errors and the noise failure mode and between the class of spurious errors and the outliers failure mode. The remaining four failure modes can be seen as specializations of the systematic errors class.

#### 3.2.3. Mitigation Techniques

Regarding mitigation techniques to address faults and respective value errors, we make a separation between what can be done at the sensor level and what can be done at the distributed system level, namely within the application that uses the sensor data, possibly exploiting additional sources of information. Considering an individual sensor, it is possible to use dependability techniques to prevent or tolerate the occurrence of faults and achieve an improved behavior, possibly even removing some failure modes. This can be described as a “basic quality improvement”, and in what follows, we describe two basic techniques that are usually carried out to achieve this objective: calibration and measurand reconstruction. The general approaches for improving the quality of data in WSN monitoring applications are then covered in Section 4.

Commonly, calibration is defined as a test under specific conditions in which pre-determined known values of the measurand are given to the transducer and the corresponding outputs are recorded. In a formal way, calibration consists of defining a function f(r,β) that, along with a set of selected device parameters β∈R, will translate real sensor output *r* to the intended output r*.

Calibration actions are required every time a sensor is deployed in a different environment, as the physical measurement elements must be adjusted or even dedicated to the monitored device or process, providing at the start a reduction of measuring uncertainty and minimal interference with sensor functions. However, periodic calibrations are also needed, since during the operation, we can assist the change of conditions with respect to those known during the calibration process and to the impact of various external factors that could be absent in the laboratory calibration conditions. These factors can be the base cause of many errors and should hence be continuously re-evaluated. For instance, in aquatic sensors, offset and drifting errors are related to the accuracy range becoming unbalanced, which is solvable by recalibration. This is done off-field (removing the sensor of the monitoring environment and recalibrating it in a container with water in controlled conditions), with potential data loss if no redundant way of collecting sensor data is available, and with re-deployment costs. It can also be done in the field, which is a time-consuming task with sometimes difficult conditions and, especially, exposing the calibration process to environmental factors that may affect the calibration accuracy.

As alternatives to manual calibration, two generic options can be considered: factory sensor calibration, with the advantage of reducing the time consumption efforts of the initial manual process, but not completely eliminating the problems mentioned before; and auto or self-calibration, enabling sensors to monitor themselves and recalibrate using a reference. This latter option, which, being adaptive, is potentially better for dealing with varied and even unpredicted misbehavior, is designated as measurand reconstruction or sensor compensation.

Auto-calibration refers to methods aimed at diminishing the effect of the disturbing parameters in the input/output features of sensors. Preferably, the transduced value must have a direct relation with the measurand, which should not be sensible to past information, interfering environmental factors, noise, error gain, etc. To try to compensate all of these disturbances, numerical techniques have to be used. These techniques are applied after the transformed signal has been quantified, through digital signal processing that must transform the sensor output signal (r*) into a corrected value ($\hat{r*}$).

Several auto-calibration techniques have been used with relative success, for instance exploiting statistical regression based on a priori knowledge [27] or artificial neural networks [28,29]. In the statistical regression approach, the goal is to determine the polynomial approximation to the characteristics of the sensor. In the artificial neural networks (ANN) approach, the inputs are the measurements, and the ideal outputs are the measurand. This model inversion is the reason why it is called measurand reconstruction. Other machine-learning algorithms have also been applied, such as Kalman filters [30] and support vector machines [31], especially in order to overcome the ANN disadvantages: neural network training may not converge to the global optimum, and training may need to be repeated several times, which will be prejudicial with respect to the computational cost; and the poor generalization capabilities that may arise from insufficient data, from over- or under-training or from under- or over-fitting.

### 3.3. Communication Faults in WSNs

When connecting individual sensor nodes in a wireless sensor network, additional faults affecting sensor data can be introduced by the network. In this subsection, we focus on the main kinds of network faults that may affect the quality of sensor data in order to achieve a reliable network operation, specifically considering faults in the time domain and faults in the value domain.

In the time domain, a crash, omission or delay faults could occur. Crash faults (for instance of the radio subsystem in a sensor node) lead to data absence and can only be mitigated with redundancy (e.g., a dual-radio system). Omissions correspond to missing sensor readings due to lost messages. They can be prevented by enforcing communication reliability, for instance based on message retransmission. However, reliable communication protocols are not very common in WSNs due to the additional resources (namely energy) they require. Therefore, omissions do happen in sensor networks and for the most part emerge because of sensor failures and packet losses. Heavy packet loss and asymmetric links occur frequently in WSNs [32,33], for instance due to signal strength fading and intermittent or continuous environmental interference (e.g., wind or rain). Absent values influence the outcome of any query over sensor readings. The resulting inaccuracies can be critical as in in-network processing and aggregations [33,34,35]. Several solutions have been suggested to tolerate these types of errors such as masking lost values through redundant information or estimating using past values [34]. Although this problem has been studied and solved in many applications, one must be aware that it is impossible to fully avoid omissions. Finally, delay faults are only relevant when the correctness of the application depends on the timeliness of sensor data. This is typically the case in real-time control, where the temporal validity of sensor data is bounded [36]. Sensor data become useless after a certain amount of time due to not reflecting the present reality with sufficient accuracy, possibly leading to system failures if used in the control process. Existing solutions to avoid timing failures are based on techniques from the real-time area, namely seizing the needed resources and using synchronized clocks to timestamp data and discard the outdated data. The existence of redundant sensor nodes can also be explored, to avoid missing important events.

In the value domain, a communication fault is translated into a message corruption. However, communication protocols typically incorporate data integrity verification mechanisms that allow the detection of corrupted messages, discarding those messages and hence transforming value faults into omission faults. Therefore, the only chance that received data do not correspond to what has been sent is when some part of the communication stack in the sending or receiving node (or both) is affected by an accidental fault not covered by the integrity verification mechanisms or when it has been intentionally corrupted. In fact, WSNs and sensor nodes can be subject to attacks that may significantly affect the quality of sensor data, among other consequences for the application. Therefore, in critical applications, it is important to deploy security techniques to avoid attacks or to mitigate their effects. These security techniques are, however, outside the scope of this survey.

## 4. Solutions for Dependable Data Quality

Several methods have been proposed in the literature to improve the quality of sensor data. Our focus is on solutions to mitigate the negative effects of faults on data quality. The ones that are applicable at the sensor level to mitigate data errors at the sensor interface have already been addressed in Section 3.2. In this section, we discuss what can be done at sink or processing nodes. We start by identifying and characterizing the three different forms of redundancy that may be explored for dependable data quality. They are related to the available sources of information, to which data analysis and processing techniques can be applied: (a) single sensor data stream, (b) multi-sensor data streams or (c) multi-source data streams.

Then, and given our focus on dependability aspects, we present a taxonomy for dependability-oriented data quality in WSNs. We identify the relevant dimensions to reason about dependable data quality, classifying the options within each of these dimensions. In this exercise, we introduce dependability-related categories concurring with the goal of estimating the quality of sensor data. In most cases, WSN-based monitoring systems address concerns (sometimes implicitly) of improving the quality of data, but not of estimating the achieved quality. The resulting systematization underlies the survey on concrete techniques for data processing, further ahead in the section.

### 4.1. Exploiting Redundancy

Redundancy is a fundamental dependability technique to achieve reliability, availability and even improved performance. Therefore, WSN applications naturally exploit the existence of multiple sensor nodes and the spatial redundancy they offer. In fact, if information relative to a certain environmental process is collected through several sensors, then it is possible to apply a range of data processing techniques to fuse the multiple data streams (from the different sensor nodes). This approach permits obtaining the resulting data with more quality, masking possible faults affecting data provided by some of the nodes. In sensor networks, it is also possible to exploit value redundancy [8] for improving the quality of data. This redundancy is offered, for instance, by environmental models describing the monitored dynamic process or setting limits to the static or dynamic attributes of this process. Finally, if sensor data from multiple sensor nodes cannot be correlated, then it is still possible to exploit a form of temporal redundancy. This temporal redundancy is intrinsic to continuous transmission, in a single flow, of data samples that can be correlated over time.

#### 4.1.1. Spatial Redundancy

The techniques aimed at exploiting spatial redundancy in WSN-based applications are known as sensor fusion techniques. Sensor fusion deals with sensor data from sensors in the same monitoring area. Through processes of comparison, combination and/or smart voting schemes, it may be possible to detect faulty behaviors, erroneous information and derive a corrected observation from the remaining (considered correct) data samples [37,38,39].

Sensor fusion is realized by employing a collection of techniques, such as classical Bayesian, Dempster–Shafer inference, artificial neural networks and fuzzy logic. The less mature techniques are dominated by heuristic and ad hoc methods. The major algorithm categories and techniques are discussed in Section 4.2.1 and Section 4.2.2.

Sensor fusion is very useful in several situations, in particular in the following: (a) when some sensors measure correctly the intended phenomena, but others do not, due to failures; (b) when all sensors measure correctly, but some respond to a different phenomenology; (c) when the data of a sensor may be masked or counter measured with respect to one sensor, but not to another; (d) when one sensor may be blocked or unable to measure, but another sensor located elsewhere may have the correct data. In this case, the data from the sensor with the correct view may be combined with past information from the blocked sensor to update the overall measurements.

The work in Reference [40] categorizes multi-sensor data fusion systems regarding what is observed by several sensors. Data fusion can take place:

1. across sensors when several sensors observe the same variable; for instance, when the temperature of a particular object is monitored by a set of temperature sensors;
2. across attributes when sensors observe several quantities related with one event; for instance, when measurements of water temperature and water conductivity are combined to define the water salinity;
3. across domains when sensors observe one specific attribute in several places. An example is when sensors in different places measure the temperature and the measured values are somehow correlated.
4. across time when new readings are fused with past data. For example, historical information from a former calibration can be incorporated to make adjustments on current measurements. Note that this is a particular case that applies to systems with single sensors, which we specifically discuss later as a form of temporal redundancy.

The work in Reference [41] provides a slightly different classification of a multi-sensor data fusion system, which partially overlaps with the previous classification. They consider that sensor fusion can be:

1. competitive when every sensor conveys an autonomous reading of the same variable. The purpose of this type of fusion is to diminish the effects of uncertain and incorrect monitoring. Competitive fusion corresponds to sensor fusion across sensors, in the terminology of [40];
2. cooperative when the data measured by many autonomous sensors is utilized to infer information that would not be accessible through each of the sensors. This corresponds to sensor fusion across attributes;
3. complementary when sensors are not directly dependent, but might be merged with the specific goal of providing a more comprehensive view of what the network is trying to observe. Thus, complementary fusion can assist in solving the incompleteness problem. This category does not entirely match the categories by [40]; it is closer to sensor fusion across attributes, but the idea is not to extract information, but to complement it.

From the above, it is clear that data fusion can take place in many ways and for different purposes, some of which are not specifically concerned with dependability issues, but rather functional issues. This is the case of cooperative sensor fusion, whose the objective is to derive new information rather than correcting the existing information.

Unfortunately, sensor fusion is not always possible. For instance, when considering monitoring activities over a wide physical area, it may be better or even necessary (namely for cost-effectiveness reasons) to scatter the sensors in pre-identified points according to area dynamics expertise and local knowledge, to cover the most significant events. For instance, this is often the case when monitoring water bodies [42], because of their typically large extension and the involved complex water dynamics, requiring expert knowledge when determining the deployment locations scattered to cover the highly variable environmental dynamics. Moreover, water monitoring usually requires costly sensors [43], which makes it infeasible to have more than one in a confined area. Exploiting sensor fusion in these conditions is thus very hard or even impossible.

Even when sensor fusion can be opted as an alternative for achieving increased dependability, there are a number of technical problems that may have to be addressed. For example, when monitoring environmental processes with fast dynamics, it may be necessary that all measurements are obtained at roughly the same time [37] so that they can be correlated. However, timing aspects are hard to deal with in distributed settings, and issues like network delays or incorrect clock synchronization of sensor nodes, if not accounted for during system design, can lead to incorrect data being produced by sensor fusion algorithms. Given the real-time nature of sensor data, there is a temporal validity interval during which the difference between the measured data value and the real value is acceptable for the application. After this temporal validity interval, data become outdated and must be discarded. Therefore, data should be timestamped as soon as they are collected, and the temporal validity interval must be known at design time. This will allow setting up mechanisms to discard outdated data. The clocks of the different nodes in the system must be synchronized and the precision (the maximum difference between all of the clocks) must also be known and taken into account when deciding whether some sensor data are already outdated. Dependable sensor fusion thus requires additional design efforts, to adapt the solution to the specific application characteristics and requirements.

#### 4.1.2. Value Redundancy

While sensor fusion relies on the physical (space) redundancy provided by the existence of several sensors, it is possible to consider data fusion [44,45] as an alternative approach. It does not require physically redundant sensor nodes, but relies on the value redundancy provided by extra information, obtained by other means. The notions of sensor fusion and (multi-sensor) data fusion are often used interchangeably. In fact, data fusion can be considered a generalization of sensor fusion, when data fusion is applied to multi-sensor data. Data fusion, in general, is related to the fusion of data, no matter its source, whereas sensor fusion (or multi-sensor data fusion) describes the use of more than one sensor in a multi-sensor system to enhance the accuracy of measured data or to handle missing data.

The process of data fusion deals with the identification, association, correlation, estimation and combination of spatially- and temporally-indexed data or information from numerous inputs with the specific goal of enhancing the analysis and understanding of this information. The techniques employed for data fusion are essentially the ones referred to for sensor fusion, which are discussed below. However, from a dependability perspective, it is important to note that data fusion opens new perspectives (in comparison to sensor fusion) regarding exploitable redundancy. We refer, in particular, to two forms of value redundancy that are exploitable with data fusion:

- Signal analysis or analytical redundancy: This is used to monitor parameters such as frequency response, signal noise and amplitude change velocity among others [46]. It is a robust approach in the case of strange behavior in a controlled system. If there is a strong variability of a variable, then a sensor is categorized as faulty (or the system under monitoring has been altered). This necessarily requires some bounds to be established a priori, against which the parameters can be fused to perform the intended classification.
- Model-based redundancy: With the help of simulation/mathematical models of the monitored system, it is possible to obtain values to validate the measurements. The author in Reference [47] was a big promoter of this type of redundancy, where the system model calculates the measured variable, and then it, is compared to the sensor measurement.

One potential difficulty in applying model-based redundancy is defining relevant and accurate models. The problem becomes even more difficult when these models characterize physical processes that change over time, which is often the case when monitoring environmental systems. Forecasting modeling techniques include simulation, estimation and syntactic methods [48]. Simulation is used when the physical characteristics to be measured can be accurately and predictably modeled. These models can be used in all types of scenarios, but most studies present examples based on terrestrial (indoor) applications [49], whereas the theme of the work herein concentrates on the complexity of the aquatic environment (e.g., water circulation). It is for this exact reason that current aquatic systems do not support real-time model-based data fusion [50]. Ideally, at run-time, a forecasting model represents a reference to validate the sensing data, which can also be applied for optimization and planning [51].

#### 4.1.3. Temporal Redundancy

In WSN applications, sensor nodes continuously send new measurements of the monitored network, typically in a periodic way, to satisfy the temporal accuracy requirements of the application.

The sequential measurements arriving at the sink or processing node constitute a time series to which data processing techniques can be applied with dependability objectives. In other words, if past measurements are considered historical data, then sensor fusion techniques can be applied to fuse the historical data with the current measurement. For instance, it is usual that noise reduction techniques are applied to single data streams, as a preliminary data enhancement step before any other data processing algorithms are applied. Outlier detection techniques [52] are also commonly applied to single data streams, detecting a faulty measurement when it deviates too much from the recent measurement history. Given the deviations caused by intrinsic noise and complex failure modes affecting the transducing process [53], choosing the adequate margins to achieve accurate outlier detection is usually a difficult problem. One approach to this problem is to use detection patterns rather than thresholds, applied to the incoming data stream. This approach allows detecting other phenomena, in addition to or instead of outliers [54]. Interestingly, outlier detection is a problem common to several areas including network intrusion, fraud detection, performance assessment and weather forecasting, among others [55].

The identification of outliers contributes to improving the data fusion processes and hence the quality of the resulting data. If performed by intermediate nodes, it may also contribute to enhancing the network performance by preventing the transmission of messages containing outliers (thus transforming outlier faults into omission faults, possibly a good strategy in systems with redundant information sources).

We note that temporal redundancy and value redundancy strategies, as described here, can be combined with spatial redundancy in a single system.

### 4.2. A Taxonomy for Dependability-Oriented Data Quality in WSNs

**Figure 4:**
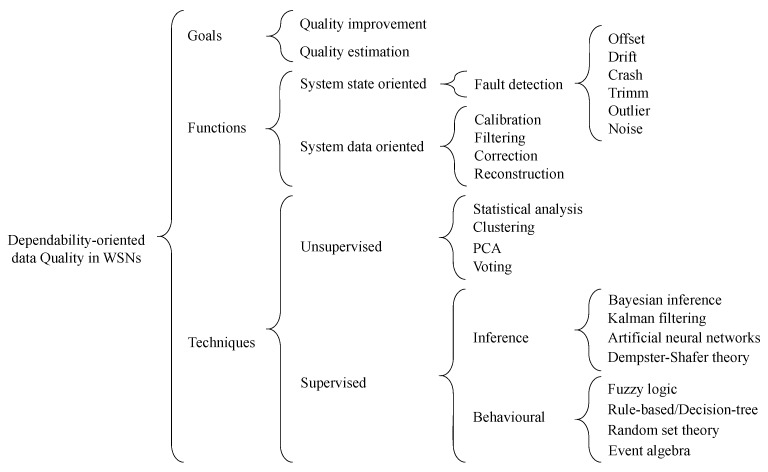
Schema of the categories of solutions for dependable WSNs.

To help the reader understanding the main dimensions, aspects and techniques that are related to the problem of achieving data quality and dependability in WSNs, we provide in Figure 4 a schema with a tree-like organization of the relevant taxonomy. Note that the redundancy approaches presented earlier serve as a base for the application of the techniques described ahead.

We consider three main dimensions that are relevant when addressing the problem of data quality and dependability improvement: goals to be achieved, functions to be performed and techniques to be applied.

We identify two distinct goals. The first consists of improving the quality of data, which is the most common in WSN applications that aim at satisfying non-functional requirements (often not explicitly specified), like reliable or safe operation. The second goal is less common. It consists of estimating the quality of data to enable assessing if non-functional requirements are satisfied. Although it may not be easy to explicitly define these requirements, the advantage is that it becomes possible to define mechanisms to mitigate the negative effects of deviations from the specification. For instance, users can be notified that the application is not working properly, or the application may be stopped in a fail-safe state instead of performing some unsafe operation.

To meet these goals, it is necessary to execute specific functions, which we classify into two categories: state oriented and data oriented. State-oriented functions are meant to evaluate the health of system components, in particular sensors (or sensor nodes), on the assumption that this health is affected by faults. Several fault detection functions are thus considered, to deal with the different failure modes identified in Section 3.2. These functions are important to both improve and estimate the quality of data, respectively by providing information that allows differentiating good and bad information sources in sensor fusion processes and by allowing distinguishing the quality of results obtained with source components in different health conditions. Data-oriented functions include all those that are meant to process sensor data, namely (but not exclusively) to calibrate, filter, correct or reconstruct data that are affected by faults. Calibration performs an automatic adjustment of values, for instance to compensate the effect of an offset. Filtering can be used to remove outliers or noise effects. Correction allows modifying values, for instance when it is know that they are drifting from the real values or that they are trimmed. Reconstruction is helpful for instance when a value is missing or when it is removed due to being an outlier and a replacement value needs to be produced. All of these functions are meant to improve the quality of data, rather than estimating this quality. They can be combined with each other and also with state-oriented functions, for better results concerning data quality improvement.

There is a vast range of techniques and specific algorithms that may be employed to process sensor data and perform the mentioned functions. In this survey, we go through the main ones, providing illustrating references, and considering the two broad categories of supervised and unsupervised techniques. No matter the function to which it contributes, when a technique requires model training and training datasets, it is characterized as a supervised learning technique. In this category, the constructed and trained models are used at run-time to classify data, estimate new values and correct existing data, among other. On the other hand, unsupervised techniques are characterized by directly inferring the possible relations between data, without the need for a correcting model output reference.

In the following sections, we include examples to help the reader understanding that for a given problem involving data quality issues, it may be possible to use multiple solutions or techniques. For instance, in [56] Kreibich et al. present two solutions for the evaluation of sensor-fusion quality in an industrial WSN that suffers from temporary losses of data and interferences in data streams, using fuzzy logic and Dempster–Shafer theory. Moreover, the authors mention that other techniques could be used (such as Bayesian, Kalman filter, artificial neural network or voting fusion).

#### 4.2.1. Supervised Techniques

Since data fusion is a concept that exists in works dated from the 1980s until now, many authors present data fusion taxonomies for detection, classification and identification algorithms [45,57,58,59]. These are low-level processing algorithms that can be applied in sensor nodes of a WSN. The goals here are to detect if an object is present, to classify the object and to identify it as accurately as possible.

Within the supervised techniques, we group the major algorithm categories into feature-based inference techniques and techniques based on behavioral models, as illustrated in the scheme of Figure 4.

Feature-based inference techniques achieve information mapping through classification or detection. An example is the use of statistical knowledge about an object or information about its features, as a means for its identification. These techniques can be further partitioned into several classes. In the following paragraphs, we will refer to some of the most frequently-used techniques, namely parametric such as Bayesian inference, Dempster–Shafer evidential theory (DST) and Kalman filters, and artificial neural networks (ANN), which is a well-known information theoretic technique. We note that there are many other machine learning techniques that may be of use, such as entropy-measuring techniques, pattern recognition, parametric templates, figures of merit, whose description falls out of the scope of this survey (we refer the interested reader, for example, to Reference [45], for further details on feature-based methods for information fusion in sensor networks).

Bayesian inference techniques use likelihood models applied to collected data to make deductions about observed quantities and even gain insights about quantities that have not been observed. Bayesian inference is used to solve the problem of efficient data gathering in sensor networks. The work in Reference [60] used this approach in a temperature and pressure sensor network composed of 500 nodes, to solve the problem of missing data, and to infer that missing information. The work in Reference [61] used a Bayesian-network-based approach to detect global outliers in an environmental monitoring network. Bayesian inference is a computationally-complex process, in which learning the classification model can be challenging, if there is a large number of correlations in the WSN.

The difficulty and uncertainty included in integrating sets of data gathered from numerous sources promoted the development of alternatives to Bayesian inference. Among them, Dempster–Shafer theory (DST) has turned out to be one of the more considered [62,63], for the most part because of the fundamental Dempster’s combination rule [64]. The biggest benefit of this method is the simplicity of consolidating possibly contradictory evidence, independently of whether it was collected as direct or indirect data. DST adapts better to the situations than the Bayesian approach as no former probabilities must be presumed regarding the potential node behavior, and acceptance of a theory does not define rejection of the contrasting proposition, which allows handling contradictory indications quantitatively. In addition, Reference [65] studied a DST approach to evaluate sensor nodes misbehavior.

Kalman filtering is a well-known estimation-based approach to solve data quality problems in WSNs. One recent example [66] presents an algorithm to correct rough and missing information grounded on Kalman filtering to surpass the issue with querying faulty information and to enhance the exactness of data in a 1000-node WSN in a synthetic environment. Another example is presented in Reference [67], in the context of an aquatic monitoring application, in which Kalman filtering was used with forecasting algorithms to assess the quality of the monitoring data series.

Artificial neural networks (ANN) are hardware or software systems that need a training process consisting of mapping input information to target values or classes. The conversion of this input information into the yields is executed by artificial neurons that try to imitate the complicated, nonlinear and hugely parallel procedures that happen in natural sensory systems. ANNs have been used in WSNs for the most varied applications, many of which are related to fault-detection [68,69,70]. In consonance with the theme of the work herein, [71] presented an ANN-based approach to detect disaster events through an environmental sensor network. Additionally, [72] presents another ANN-based approach to detect biofouling events (thus, fault events) in an aquatic sensor network.

The behavioral (cognitive-based) models group encompasses techniques that attempt to imitate and mechanize the decision-making procedures utilized by human analysts. These include event algebra, rule-based systems and fuzzy logic. The latter technique is the most studied and applied, which justifies our particular attention to it.

According to [73], fuzzy set theory allows for imprecise knowledge to be mathematically treated by making it easier to represent or classify system state variable information. The use of fuzzy associative memory (also known as production rules) allows a proposition to have a membership value in a given class ranging from zero (absolutely does not belong in the category) to one (absolutely belongs in the category). An expert specifies the production rules and fuzzy sets that represent the characteristics of each input and output variable. Fuzzy data fusion application to WSNs has at least as much popularity as ANN-based fusion; therefore, its applications range from fault detection [74,75,76] to applications in industrial WSNs [77], the environment [78] and aquatic-related WSNs [79].

There are some other mathematical approaches that have been developed in recent years, which include random set theory, conditional algebra and relational event algebra [48].

Random set theory complements the existing theories of random vectors and of random functions serving as a mechanism for modeling observed phenomena, which are sets rather than precise points. It can be applied to incorporate ambiguous evidence (e.g., natural language reports and rules) and various expert system methods into multi-sensor estimation. Conditional event algebra refers to sets with one or more finitary operations defined on it that satisfy a list of axioms, whose domain consists of logical objects using a type of probabilistic calculus suited for contingency problems such as knowledge-based rules and contingent decision making. Relational event algebra is an extension of conditional event algebra where functions of probabilities formally representing single event probabilities represent actual relational events considering appropriately determined larger probability spaces, providing a systematic basis for solving problems involving pooling of evidence.

#### 4.2.2. Unsupervised Techniques

There are several unsupervised data processing techniques (Figure 4), which serve, just like supervised techniques, to perform the needed functions in WSN-based monitoring systems, like detection, filtering or correction.

Various statistical analysis methods can be used as unsupervised techniques for data processing. For instance, the work in [80] resorts to statistical analysis to identify events, recognize observation errors and predict absent measurements in ecological WSNs. The proposed method requires learning statistical distributions of differences between measurements of a sensor and those of its neighbors, as well as between sequences of single-sensor measurements. According to the author, there is a large degree of spatiotemporal correlation in scalar physical variables, which provides a spectrum of oscillations between adjoining or successive readings with little differences. Based on successive readings, it is possible to learn their distribution and then detect outliers when a reading value is lower than a determined threshold, in the statistical significance test.

Clustering techniques are quite common in WSN-based applications. The general procedure is to integrate analogous information into groups with identical comportment. Data not belonging to these clusters or belonging to a smaller cluster would be considered outliers, if this is the goal. A simple and well-known clustering algorithm is the nearest neighbor, which associates the most similar measurements. For example, the approach was used by [81] to handle unsupervised outlier detection and, in particular, to identify global-wise outliers. Every node utilizes distance similitude to locally distinguish anomalous readings and transferring those readings to the nearby nodes for confirmation. These nearby nodes will repeat this process until the entire network ultimately agrees on the overall anomalous readings. The downside of this method is the lack of scalability to large-scale networks. The most used method to measure the similarity between two data instances is the Euclidean distance. For instance, this is used in [82] in the context of target classification in a multi-channel seismic network.

The spectral decomposition-based approach aims at defining standard behaviors in the data by utilizing principal component analysis (PCA). PCA allows decreasing the magnitude of an information set in which there are many interrelated variables, while holding as much as could be expected of the variety present in the set. The work in Reference [83] proposed a PCA-based method to address the data integrity arising from the imprecision triggered by faulty sensor nodes. The method requires a model of the standard behavior to be built a priori, by selecting appropriate principal components (PCs), and allows the detection of outliers.

Voting methods are useful to fuse information from several sensors, particularly when applied to detection and classification declarations from multiple sensors. These declarations are treated as votes, to which majority, plurality or decision-tree rules are applied to obtain a result that is more dependable than what would be obtained with a single sensor output [48]. This allows, for instance, masking false alarms when the sensors are used to detect the occurrence of some event, thus preventing premature reactions or countermeasures. In this sense, voting methods are also appropriate for fault-detection, to decide which node is the faulty one [84,85]. Finally, they are used in several other application contexts, such as WSN security [86] and sensor faults in on-body sensor networks [87].

## 5. Conclusions

Assuring the quality of sensor data is important in WSN-based monitoring applications. In the last decade, this dependability aspect has been explicitly or implicitly addressed in many works, notably by exploiting the redundancy provided by the multiple sensor nodes typically existing in a WSN. Various specific problems need to be addressed when aiming at a dependable WSN-based monitoring solution, from ensuring the reliability of the transducing process to achieving a correct interpretation of data collected from several correlated sensors.

In this paper, we present an encompassing perspective of the several facets of the problem, focusing on dependability aspects specific to individual sensors, to the network that interconnects the sensor nodes and the processing nodes and to the processing tasks that are performed within the processing nodes. This separation of concerns allows one to: (a) clearly expose the possible causes of data quality loss from the source to the final output; (b) describe specific mitigation solutions; (c) provide a dependability perspective on what can be explicitly done to achieve improved data quality and assess this quality. Particular focus is given to the different forms of redundancy that may be exploited to achieve the dependability objectives: spatial, value and temporal redundancy. These are intrinsically related to the many sensor and data fusion techniques commonly employed, also surveyed in the paper. We provide many references on publications with theoretical and practical applications of the techniques, chosen to illustrate the multitude of options that are studied to solve directly or indirectly data quality problems. A specific outlook on data quality issues and open problems in water monitoring applications is finally given.

## Figures and Tables

**Table 1:** Advantages and disadvantages of the various displacement effects [23,24,25].

Displacement Effects	Advantages	Disadvantages
Resistance	Versatile; inexpensive; easy-to-use; precise.	Limited bandwidth; limited durability.
Induction	Robust; compact; not easily affected by external factors.	A significant part of the measurement is external, which must be well cleaned and calibrated.
Capacitance	Low-power consumption; non-contacting; resists shocks and intense vibrations; tolerant to high temperatures; high sensitivity over a wide temperature range.	Short sensing distance; humidity in coastal/water climates can affect sensing output; not at all selective for its target; non-linearity problems.
Piezoelectricity	Ideal for use in low-noise measurement systems; high sensitivity; low cost; broad frequency range; exceptional linearity; excellent repeatability; small size.	Cannot be used for static measurements; high temperatures cause a drop in internal resistance and sensitivity (characteristics vary with temperature).
Laser	Ideal for near real-time applications; low uncertainty and high precision in the measurements.	Weather and visual paths affect the sensor when measuring distance or related variables.
Ultrasonic	Independent of the surface color or optical reflectivity of the sensing object; excellent repeatability and sensing accuracy; response is linear with distance.	Requires a hard flat surface; not immune to loud noise; slow measurements in proximity sensors; changes in the environment affect the response; targets with low density may absorb sound energy; minimum sensing distance required.
Optical encoding	Inherently digital (which makes the interface easy for control systems); fast measurements; long durability.	Fairly complex; delicate parts; low tolerance to mechanical abuse; low tolerance to high temperatures.
Magnetic	Non-contacting; high durability; high sensitivity; small size; output is highly linear.	Very sensitive to fabrication tolerances; calibration needed after installation.
